# Microstructure Evolution during Dissimilar Friction Stir Welding of AA7003-T4 and AA6060-T4

**DOI:** 10.3390/ma11030342

**Published:** 2018-02-27

**Authors:** Jialiang Dong, Datong Zhang, Weiwen Zhang, Wen Zhang, Cheng Qiu

**Affiliations:** National Engineering Research Center of Near-net shape Forming for Metallic Materials, South China University of Technology, Guangzhou 510640, China; 201610100603@mail.scut.edu.cn (J.D.); mewzhang@scut.edu.cn (W.Z.); jack_eei@scut.edu.cn (W.Z.); cqiu@scut.edu.cn (C.Q.)

**Keywords:** dissimilar friction stir welding, mechanical properties, microstructure evolution, dynamic recrystallization

## Abstract

In this work, the dissimilar joint of AA7003-T4 and 6060-T4 alloy has been produced by friction stir welding (FSW). The microstructure was examined by optical microscope (OM), electron back scattered diffraction (EBSD), transmission electron microscopy (TEM), and the mechanical properties of the joint were investigated. It is demonstrated that sound dissimilar joint can be produced through FSW. In the nugget; precipitations dissolve into the matrix and η′ reprecipitate subsequently; and the elongated aluminum grains are replaced by fine and equiaxed grains due to dynamic recrystallization (DRX). In the heat affected zone (HAZ), coarse β′ and η precipitates are formed and the aluminum grains are coarser as compared to the base materials. In the thermo-mechanical affected zone (TMAZ), equiaxed and elongated grains coexist due to incomplete DRX. The ultimate tensile strength of the dissimilar joint is 159.2 MPa and its elongation is 10.4%. The weak area exists in the HAZ of 6060 alloy, which is placed in the retreating side during FSW. The correlations between the microstucture and mechanical properties of the dissimilar joint are discussed.

## 1. Introduction

As typical light alloys with high specific strength, aluminum alloys are widely used in industry due to their excellent physical and mechanical properties, good workability, and ease of recycling [1,2]. The application of aluminum alloys are quite important in aerospace and automobile industries, since light-weight design can bring many benefits, including energy saving, emission reduction, and performance improvement. 6XXX and 7XXX are heat-treatable aluminum alloys, in which precipitation hardening is the main strengthening mechanism [3,4,5]. 6XXX series alloys have medium strength and good formability, while 7XXX series are well-known as typical high-strength aluminum alloys. In many fields, the joining of these two series alloys needs to be solved. For instance, the combinations of 6XXX and 7XXX alloys can be used in aerospace structures [6] and automobile parts (such as bumper beams [7]). However, traditional fusion welding technologies, such as tungsten inert gas welding (TIG) and metal inert gas welding (MIG), are not appropriate for the dissimilar joining of 6XXX and 7XXX alloys. In fact, 7XXX aluminum alloys are generally considered as un-weldable alloys since their weldability are relatively poor in fusion welding processes [6,8,9].

Friction stir welding (FSW), which was firstly invented by TWI in 1991, is an efficient joining technology for metallic materials [10,11]. Since the entire process of FSW is completed below the melting point of the alloys, many defects that are caused by solidification, such as gas pores, segregation, and hot cracking, can be avoided. It has been reported extensively that high quality joints of 7XXX aluminum alloys can be produced by FSW [12,13]. Consequently, FSW provides a feasible method for the dissimilar joining of 6XXX and 7XXX alloys.

Recently, some investigations on dissimilar FSW of 6XXX and 7XXX alloys have been reported [6,14,15,16,17,18,19,20,21]. Rodriguez [16] studied the mechanical properties of dissimilar FSW of 6061 to 7050 aluminum alloys, the ultimate tensile strength (UTS) of the joints was 50% of the 6061-T6 base material (BM), and the maximum elongation was 8%. Bahemmat [6] studied the microstructure and the mechanical properties of dissimilar FSW of AA6061-T6 and AA7075-T6, and the UTS of the joints is 72% of 6061-T6. As compared to the FSW joints of 6XXX alloys, the joint efficiency of dissimilar 6XXX to 7XXX joints needs to be improved. Since the mechanical characteristics and flow behaviors of the 6XXX and 7XXX alloys are different, the processing design for 6XXX/7XXX dissimilar FSW is difficult to obtain optimized parameters.

Microstructure evolution during FSW is relatively complicated since severe plastic deformation and dynamic recrystallization (DRX) may take place in the welding zone, i.e., the nugget. As to the precipitation hardening alloys, the transformation of the strengthening phases should also be concerned. Olea [22] and Sato [23] found that the strengthening phases in 6XXX alloys would completely dissolve in the nugget to form supersaturate solid solutions during FSW. However, in FSW of 6056 alloys, Cabibbo [24] found that the coarse equilibrium β phase existed in the stirred zone (SZ) and thermo-mechanical affected zone (TMAZ). In FSW of 7050 alloy, Reynolds reported that partial or complete dissolution of the precipitates took place in the nugget [25]. Since the thermal history may play different roles on 6XXX and 7XXX alloys during FSW, precipitation evolution behavior, and its effect on the mechanical properties of 6XXX/7XXX dissimilar joints need to be investigated in detail. Until now, this topic is still not fully understood.

In this paper, dissimilar FSW joint of AA7003-T4 and 6060-T4 alloys were produced. The material flow and microstructures in different areas of the joint were investigated. The strengthening phases in the dissimilar joint were analysed in detail. Based on the experimental results, the relationship between the microstructures and the mechanical properties of the dissimilar joint were investigated.

## 2. Materials and Methods

The base materials used in this study are extruded plates of AA6060 and AA7003 Al alloy in T4 temper condition, and the chemical compositions, which are measured by direct-reading spectrometer (ARL4460, Thermo Scientific, Switzerland), are shown in Table 1. The thickness of the plates is 4.5 mm. The plates are cut into dimensions with 150 mm long and 50 mm wide. The dissimilar FSW experiments are carried out on FSW-3LM-003 equipment (FSW Technology Co. Ltd., Beijing, China), and a welding tool with a shoulder of 18 mm in diameter, a threaded conical pin of 4 mm in length and 6 mm in root diameter is used. Before welding, the surfaces of the plates are polished with 1000 grit size emery papers to remove the oxide layer. The welding direction is parallel along the extrusion direction of the plates. 7003 plate is placed on the advancing side (AS) and 6060 is placed on the retreating side (RS). The welding process is accomplished at a rotational speed of 1000 r/min and a welding speed of 40 mm/min. The angle between the tool axis and the normal direction of the plates is selected as 2.5°.

After FSW, the specimens are cut perpendicular to the welding direction for the microstructural observation and the mechanical properties tests. The specimens are etched in the Keller reagent after being polished by diamond paste. Macrostructure and microstructure of the joint are examined by optical microscopy (OM, Keyence, VHX-600, Osaka, Japan) and SEM (Nova Nano430, FEI, Hillsboro, OR, USA). In SEM, SE mode is used with an acceleration voltage of 15 KV, and the best working distance is 10 mm. Grain structures of the experimental materials are analysed by electron back-scattered diffraction (EBSD, S-3400N, Hitachi, Tokyo, Japan), equipped on SEM with a tilt angle of 70 degree. EBSD samples are prepared by ion-etched method. Precipitates are observed by transmission electron microscopy (TEM, Gatan, PIPS-691, Pleasanton, CA, USA), and thin TEM samples are prepared via an ion-miller.

Vickers microhardness distribution is measured on the cross-section of the joint under a load of 100 g and a dwelling time of 10 s. The tensile tests are conducted on hydraulic universal material testing machine (Instron, Shanghai, China) at room temperature under a displacement control mode at a rate of 1 mm/min. Tensile fracture morphologies are also examined by SEM (Nova Nano430, FEI, Hillsboro, OR, USA).

## 3. Results

### 3.1. Microstructure of the Base Materials

**Figure 1:**
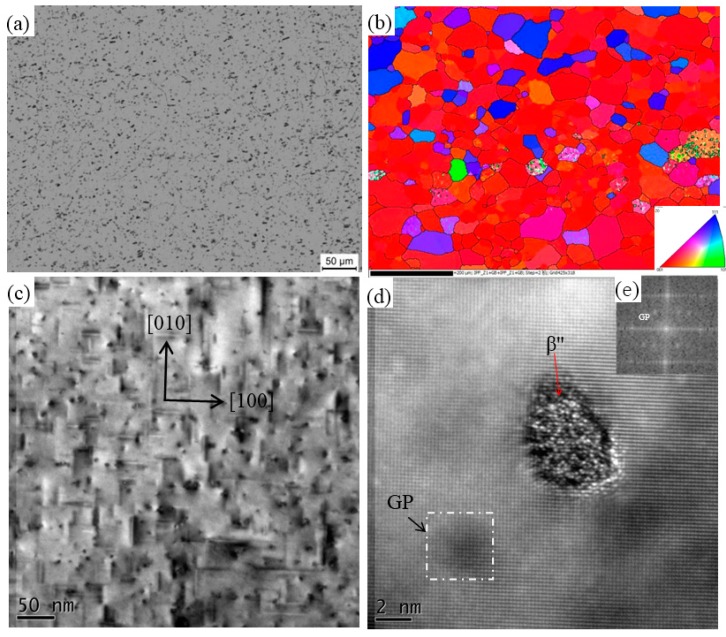
Microstructures of 6060-T4 (**a**) optical microscopy (OM); (**b**) Orientation image map (OIM); (**c**) transmission electron microscopy (TEM); (**d**) high-resolution transmission electron microscopy (HRTEM); and, (**e**) Fast Fourier Transform (FFT) of the marked GP zone.

Microstructures of BM 6060-T4 are illustrated in Figure 1. Figure 1a,b show that the 6060-T4 are composed of bulk grains and that the average grain size is about 30 μm. Fine precipitates are distributed homogeneously in the matrix, as shown in Figure 1c. Further analysis illustrates that these needle-like phases are alongside <100> in Al matrix, with their sizes ranging from 20 to 50 nm in length, so the precipitates are confirmed as β′′ which are the main second phase of 6XXX alloys in T4 state [26,27]. Moreover, some fine and spherical phases exist in the matrix. Through high-resolution transmission electron microscopy (HRTEM) in Figure 1d, the diameter of spherical precipitate is about 4 nm and it is fully coherent with the Al matrix. According to other people’s research, this precipitates are also β′′ phase [28,29]. In Figure 1d, round dark zone pointed by arrow can also be observed, which is fully coherent with Al matrix. This is GP zone, which is also found in other 6XXX alloys [30,31]. It is also confirmed by the Fast Fourier Transform (FFT) shown in Figure 1e. Hence, both GP zone and β′′ exist in BM 6060-T4.

**Figure 2:**
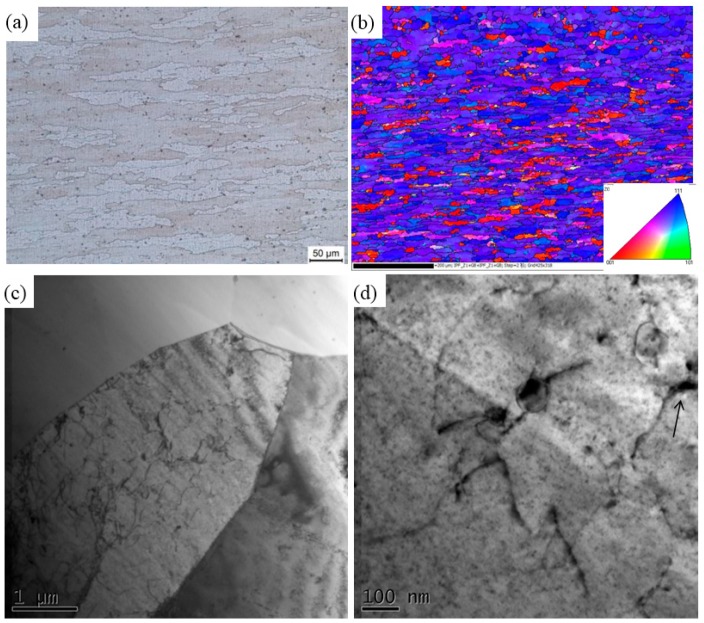
The (**a**) OM; (**b**) OIM; and (**c**,**d**) TEM micrograph of 7003-T4.

**Figure 3:**
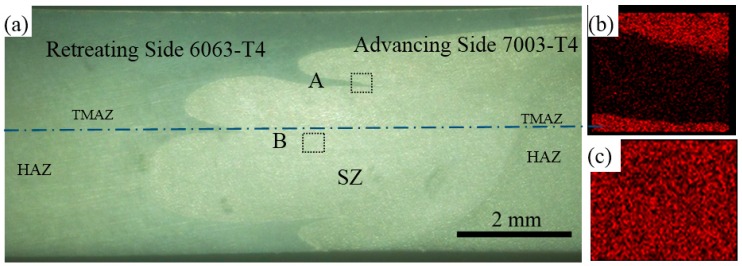
(**a**) Macrostructure of dissimilar FSW joint; EDS map of element Zn distribution in location of (**b**) A and (**c**) B.

The optical morphology, Orientation image map (OIM) and TEM of 7003-T4 are shown in Figure 2. From Figure 2a,b, it can been seen that 7003-T4 consists of elongated grains with ~35 μm in length and 7 μm in width. Figure 3c shows that relatively fine precipitates exist within grains, which are confirmed as metastable η′ phase in the high magnification TEM (Figure 2d) [32]. The coarse precipitates pointed by arrows in Figure 2d are confirmed as AlFeMnSi impurity phase.

### 3.2. Macrostructure of the Dissimilar FSW Joint

The macrostructure of the dissimilar FSW joint is shown in Figure 3a. Sound and defect-free joint is obtained. In the top region of the nugget, a bulk of 6060 alloy is extruded from RS toward AS of the weld. In the middle region of nugget, it is mainly composed of 7003 alloy. It is pointed out that in dissimilar FSW of 6XXX and 7XXX alloys, the nugget is dominated by the alloy that is placed on the AS [15]. The sharp interface indicates that 7003 and 6060 are simply extruded into the nugget without extensive intermixing. Figure 3b,c show the EDS maps of Zn distribution in the relevant areas marked in Figure 3a. The formation of nugget is depended on the FSW tools' geography, material related position, and processing parameters [33]. Guo et al. reported that placing the harder alloy on RS could enhance the material flow in dissimilar FSW [14]. On the other hand, researches of Giraud [9] and Srinivasan [18] suggested that the harder alloys should be placed at AS. Therefore, Guo pointed out that the relative position of the dissimilar plates is possibly related to the mateirals pair [14]. In our study, by placing the soft alloy on RS, it is easier to form flawless joints since the material flow is more convenient to fill the cavity behind the pin during FSW Consequently, the setting of the dissimilar plates, i.e., 6060 in RS and 7003 in AS, is beneficial to produce sound joints. Moreover, since the majority of the nugget is composed of 7003 alloy with higher strength, the strength of the joint can also be improved.

### 3.3. Grains of Stirred Zone

**Figure 4:**
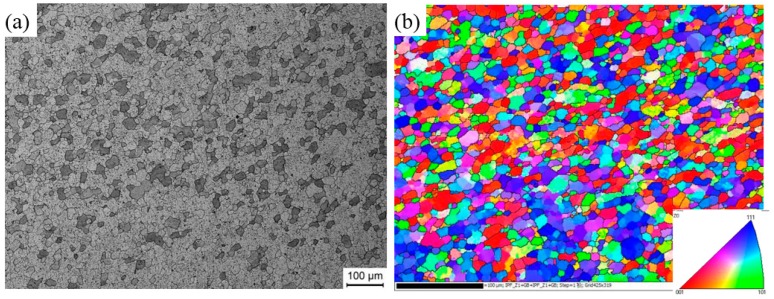
(**a**) Microstructure and (**b**) OIM of SZ of as-welded sample.

**Figure 5:**
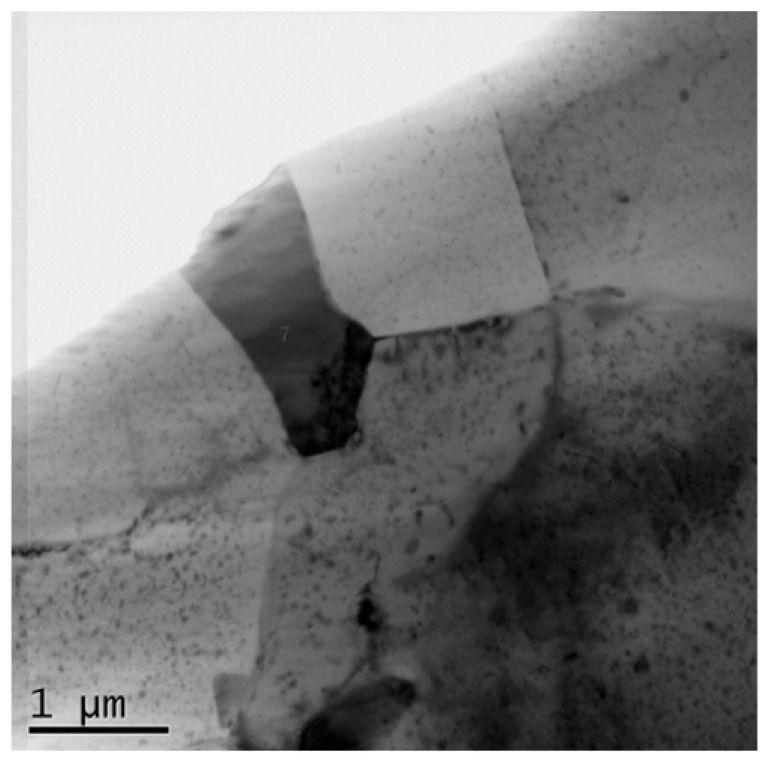
TEM of the weld nugget.

Microstructure of SZ in the middle region of the nugget are shown in Figure 4. As mentioned above, the observed zone is composed of 7003 alloy. The nugget consists of fine equiaxed grains with an average grain size of 11.1 μm. When compared with Figure 2, the alloy in SZ experiences severe plastic deformation during FSW, consequently DRX occurs and the elongated grains are replaced by equiaxed fine grains. Figure 5 shows the TEM photograph of SZ, in which the newly-formed DRX grains can be seen clearly. During FSW, continuous dynamic recrystallization (CDRX) is the main mechanism for the grain refinment of aluminum alloys [13,34].

### 3.4. Precipitates Distribution

**Figure 6:**
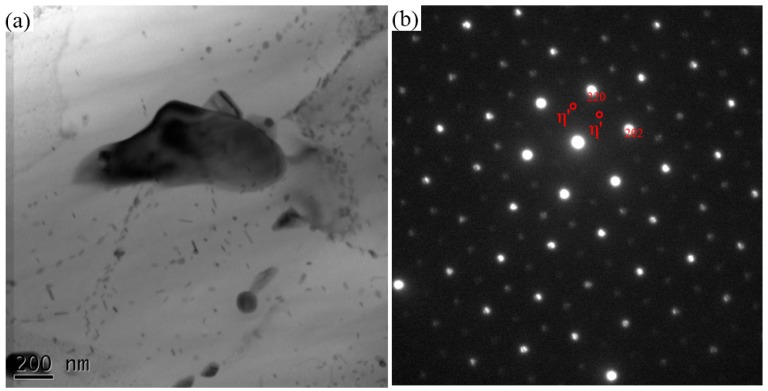
TEM of (**a**) Precipitates in the nugget and (**b**) selected area electron diffraction (SAED) pattern.

The TEM specimen is cut in the middle part of nugget, so it is composed of 7003 alloy. The result is shown in Figure 6. A coarse dark particle can be seen in Figure 6a, which is confirmed as AlFeMnSi impurity phase by EDS result. The selected area electron diffraction (SAED) pattern along [111]Al incident beam is shown in Figure 5b, which reveals the aluminum matrix and η′ precipitates [35]. When comparing the precipitates in the parent material shown in Figure 2, the AlFeMnSi particles become coarser and the number density of the fine precipitates, i.e., η′, decreases. During FSW, the precipitates in BM would dissolve into the matrix due to the high temperature and mechanically stirring effect. In the subsequent cooling, finer η′ phases reprecipitate. As Su et al. pointed out, the continually introduced (sub-)boundaries and dislocations by stirring promotes the reprecipitation of η′ [13].

**Figure 7:**
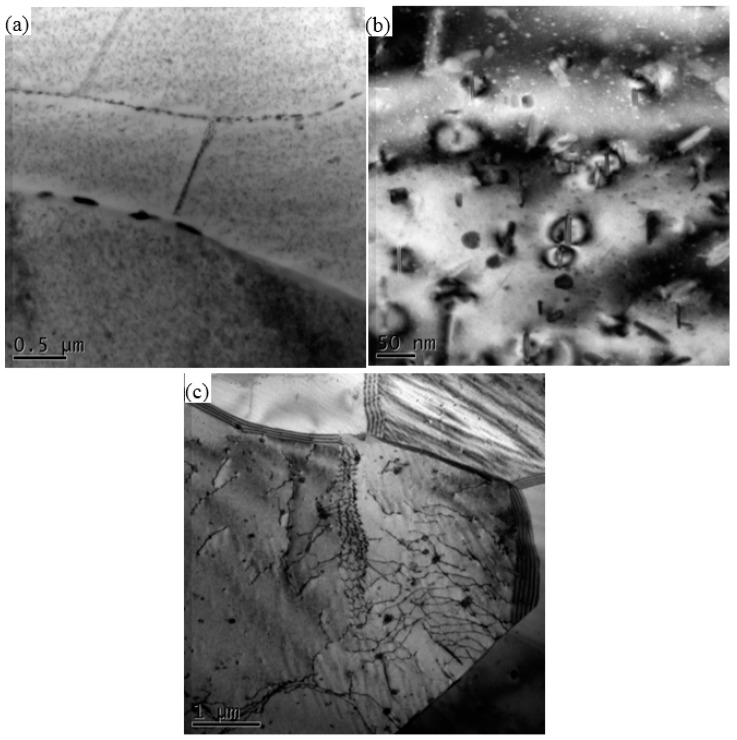
TEM of (**a**) heat-affecting-zone (HAZ); (**b**) magnification of (**a**,**c**) thermo-mechanical affected zone (TMAZ) in advancing side (AS).

The TEM result of HAZ in AS is shown in Figure 7, which illustrates that there are abundant intragranular precipitates. Some coarse precipitates and precipitate free zone (PFZ) could be observed along grains boundaries. The magnification of intragranular precipitates is shown in Figure 7b. The fine plate-like precipitates are η′ and the coarse precipitates are η. Since the material in HAZ only experience heat exposure, the precipitates dissolution is insufficient. Therefore, the precipitation density in HAZ is higher than that in SZ. The microstructure of TMAZ in AS is shown in Figure 7c. Most of the precipitates have dissolved into matrix, and only a few round particles remain in the Al matrix, dislocations around these particles are also observed.

**Figure 8:**
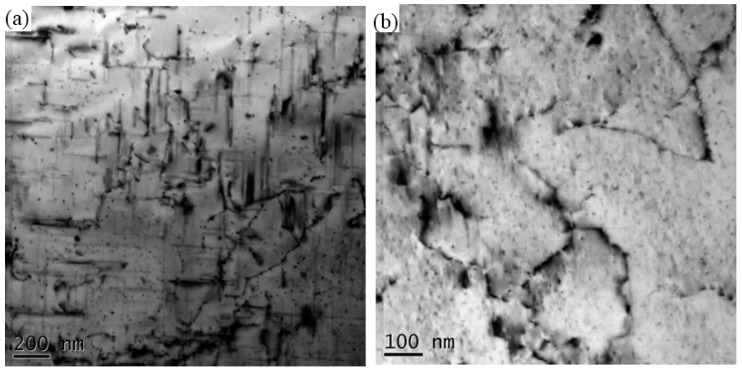
TEM of (**a**) HAZ and (**b**) TMAZ in retreating side (RS).

The TEM photographs of HAZ and TMAZ in RS are shown in Figure 8. The intragranular precipitates exhibit a needle-like shape with their length ranging from ~50 to 200 nm. When compared with Figure 1, the number density of precipitates drops greatly and the precipitates become coarser. It is considered that some β′′ precipitates are transformed into β′ in HAZ. Figure 8b shows that relatively fine precipitates exist in TMAZ. These intragranular precipitates with a size of a few nanometres are GP zone. During FSW, the precipitates in the parent material have dissolved into matrix and GP zone reprecipitates subsequently in TMAZ.

### 3.5. Mechanical Properties

#### 3.5.1. Microhardness Testing

**Figure 9:**
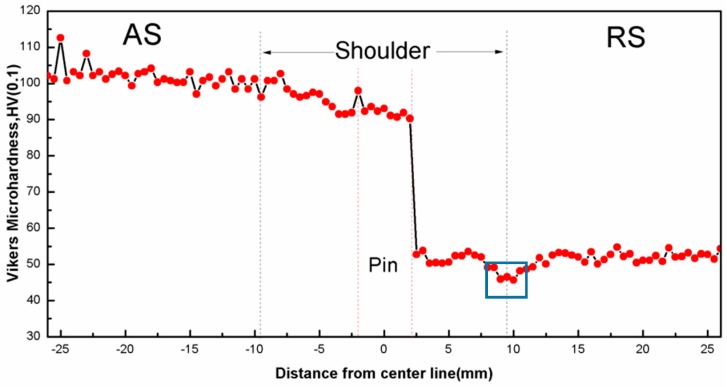
Microhardness distribution of the dissimilar joint.

Microhardness is measured along the centerline marked in Figure 3a, and the microhardness profiles of the dissimilar joint is shown in Figure 9. Because of the different microstructure and the mechanical properties of the base materials, an asymmetric microhardness distribution is obtained, which is also seen in other studies [14,15,16,17,18,19,20,21]. Since the hardness of 7003 in AS is higher than that of 6060 in RS, the hardness profile presents a feature of stair shape. The microhardness values of the nugget are a little lower than BM 7003, which is mainly attributed to the precipitates density decreasing during FSW. There is a drop of microhardness in RS, wherein the smallest Vickers microhardness value is 45, the same value of 6060-O [36]. The region that has the lowest hardness in the whole joint is located at HAZ in RS.

#### 3.5.2. Tensile Testing

**Figure 10:**
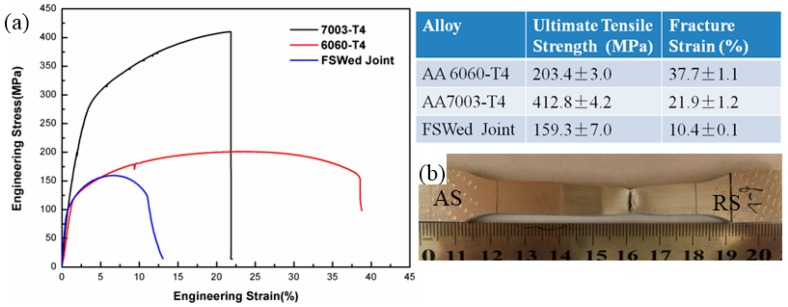
The uniaxial tensile curve and sample.

The tensile test results are shown in Figure 10, and detail mechanical properties are listed in the attached table. The ultimate tensile strength (UTS) of the joint is 159.2 MPa, which is 78.3% of 6060-T4 alloy and 38.7% of 7003-T4 alloy. The elongation of the dissimilar joint is 10.4%. A typical tensile test sample is shown in Figure 10b. Necking can be seen clearly on the failed sample, and the fracture occurs on HAZ in RS.

## 4. Discussion

### 4.1. Precipitates Evolution

6060 and 7003 alloys are precipitation hardening alloys, and the phase transformation mechanisms in various heat-treatments have been studied extensively [3,4,5]. In T4 state, both GP zones and metastable phases exist in these alloys, as shown in Figure 1 and Figure 2. Further transformations will take place in the T4 alloys, according to the thermal history in the subsequent processing, including GP zone dissolving, reprecipitation, etc. For instance, Deschamps [37] reported that GP zone in 7XXX alloy will dissolve into matrix at ~150 °C, while the GP zone dissolving temperature in 6XXX is about 190 °C [38]. The solvus of η′ and η in 7XXX alloys is about 355 and 438 °C, respectively, while in 6XXX alloys, the temperature of β′′ dissolving is about 300 [37,38]. In Figure 6, the number density of the precipitates decreases greatly and a few η′ particles and coarse impurity particles are observed.

The temperature distribution during FSW is mainly depended on the material characteristics, tool geometry and processing parameters. Dissimilar FSW of 6XXX/7XXX alloys [17,21] showed that the peak temperature was about 500 °C. Hence, a complete dissolution of the precipitates in nugget occurs during FSW. Zhang [39] found that the temperature was higher than 200 °C in HAZ 20 mm away from the weld center. When compared with other studies on dissimilar 6XXX/7XXX FSW [16,17,21], higher rational speed and lower welding speed are used in this experiment, which means longer thermal exposure and larger deformation are introduced during FSW. Hence, it is considered that the thermal-mechanical conditions in this study will facilitate the metastable phases in the parent materials to dissolve in the nugget. Reprecipitation takes place in the cooling stage after FSW.

**Figure 11:**
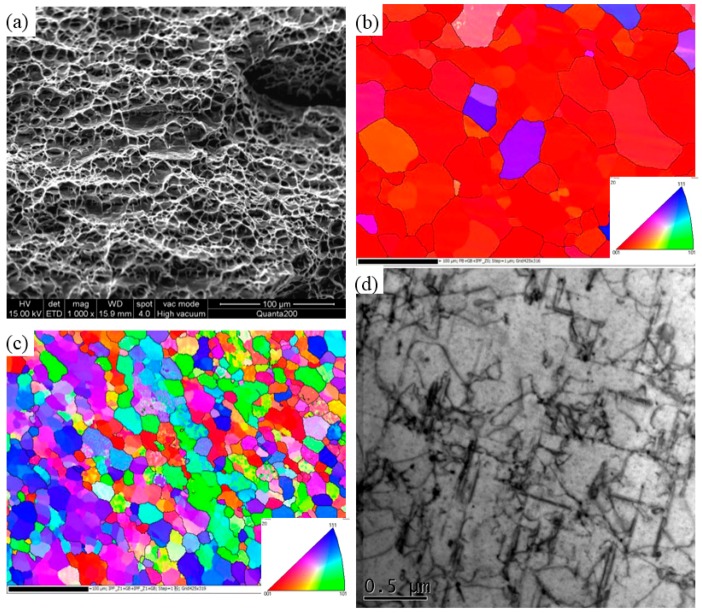
(**a**) Fracture surface and (**b**,**c**) the OIM of HAZ, TMAZ, respectively; (**d**) TEM picture of heat-affecting-zone (HAZ) of fracture area.

When comparing Figure 5 and Figure 7c, it can be seen that the number density of reprecipitates is higher and the size is larger in SZ than those in TMAZ. Because of the friction between rotational spin and BM, the temperature in nugget is higher than that in TMAZ, and the lasting time at high temperature is longer in SZ. These conditions are beneficial for strengthening phases reprecipitation. Furthermore, Su et al. [13] have shown that the stirring effect would introduce more dislocations and (sub-)boundaries wherein reprecipitation could take place preferentially. Hence, the stirring effect promotes the reprecipitation of η′ in nugget. In TMAZ, the stirring effect is weaker than that in SZ. Consequently, finer and less reprecipitates can be seen in TMAZ. In HAZ, the local microstructures are only affected by heat. Although the temperature of HAZ is lower than those of TMAZ and SZ, it is still high enough for microstructure coarsening. From Figure 7a and Figure 8a, the precipitates in HAZ at both AS and RS become coarser. Moreover, the aluminum grains in HAZ also grow coarse, which can be seen in Figure 11.

### 4.2. Mechanical Behavior of Dissimilar FSW Joint

In 6XXX alloys, the distribution and number density of GP and β′′ determine the mechanical properties and the strengthening effect of β′′ is the maximum [40]. In 7XXX aluminum alloys, the major strengthening precipitations in Al-Zn-Mg(-Cu) are considered to be GP zone and η′ [32], and GP zone provides less contribution for strengthening than η′. The microhardness distribution (Figure 8) corresponds to the microstructure evolution that is caused by the thermo-mechanical history during FSW. The minimum microhardness area marked by box exists in RS. This region is in the HAZ, where the number density of strengthening precipitates decreases due to coarsening, as shown in Figure 11d. A tendency can be observed that the hardness increases gradually with increasing distance away from the weld center. Because of the differences of precipitates size and density, Vickers microhardness in AS is a little higher than that in nugget.

Because the strength and hardness of 7003 are much higher than that of 6060, so fracture tends to take place in 6060 side. Rodriguez [16] reported that all of the fractures occur in TMAZ or HAZ of 6XXX side. The tensile sample in Figure 9 shows that the exact fracture location is in HAZ near TMAZ in RS, corresponding to the minimum hardness region. The fracture surface is shown in Figure 11a. It is found that the fracture surface is full of small dimples, which demonstrate good ductility of 6060 alloy. Microstructures on both sides of the fracture surface are analysed by SEM and TEM. Figure 11b shows that the average grain size is 42.3 μm in HAZ and Figure 11c shows that the average grains size in TMAZ is 20.3 μm. Elongated grains can be seen in Figure 11c, which means that the DRX in TMAZ is incomplete. When compared with BM 6060-T4, precipitates along <100> Al matrix become coarser and the number density decreases in HAZ, as shown in Figure 11d. The transformation of β′′ to β′ occurs due to the heat exposure. Therefore, the coarsening of aluminum grains and precipitates are the main reasons for the weakening of HAZ, wherein the tensile fracture occurs and the lowest microhardness is observed.

## 5. Conclusions

Dissimilar joints of 7003-T4 and 6060-T4 aluminum alloys have been prepared by FSW. Microstructure and mechanical properties of the joints are studied. Based on the results, conclusions are drawn as follows.

1. By placing the soft 6060 alloy on the retreating side, sound joints are produced through dissimilar FSW. Bonding interface can be seen clearly in the nugget, and the main part of the SZ is composed of 7003, which is on the advancing side.

2. In nugget, fine and equiaxed grains are obtained due to DRX. Precipitations have dissolved into matrix and η′ reprecipitates subsequently, with the number density decreasing greatly. The impurity phase particles grow coarser.

3. In HAZ, the aluminuum grains become coarser. The transformations of metastable GP and η′ to η on 7003 side, GP and β′′ to β′ on 6060 side, have taken place, respectively. The number density of precipitates decrease in HAZ. In TMAZ, fine and equiaxed grains and elongated grains coexist due to an incomplete DRX. Finer and less reprecipitates are observed.

4. The ultimate tensile strength of the dissimilar joint is 159.2 MPa, 78.2% of BM 6060-T4, and its elongation is 10.4%. There is a weak zone in HAZ on 6060 side, wherein the lowest hardness is 45HV and tensile fracture occurs. The fracture surface shows a typical ductile fracture morphology.

## Figures and Tables

**Table 1:** Chemical composition of the experimental alloys (wt %).

Alloy	Si	Mg	Zn	Fe	Mn	Ti	Ni	Cr	Al
AA6060-T4	0.427	0.314	0.020	0.127	0.006	0.011	0.004	0.006	Bal.
AA7003-T4	0.090	0.470	5.672	0.126	0.113	0.029	0.004	0.004	Bal.
